# Prevalence and Predictors of Anaemia Among Women of Reproductive Age in South and Southeast Asia

**DOI:** 10.7759/cureus.50090

**Published:** 2023-12-06

**Authors:** Tahmina A Keya

**Affiliations:** 1 Department of Community Medicine, Faculty of Medicine, AIMST (Asian Institute of Medicine, Science and Technology) University, Bedong, MYS; 2 Department of Community Medicine, Sri Balaji Vidyapeeth Deemed to be University, Puducherry, IND

**Keywords:** south-east asian, early predictors, developing countries, women of reproductive age, anaemia

## Abstract

Background

Anaemia is a global public health issue that mostly affects children, women, and adults in low- and middle-income countries.

Aim

This study sought to evaluate the prevalence of anaemia and its contributing factors among South and Southeast Asian (SSEA) women of reproductive age.

Methods

Data analysis was undertaken utilising data from 10 SSEA countries' Demographic and Health Surveys. The link between anaemia and independent variables was established using a multivariate logistic regression model with a 95% confidence interval. To investigate the relationship between explanatory variables and anaemia, the Pearson's correlations test was carried out.

Results

In SSEA, the prevalence of anaemia among women (15-49 years old) was 50.17% (95% CI: 39.4, 61.0), ranging from 13.3% (95% CI: 2.5, 24.1) in the Philippines to 70.3% (95% CI: 59.5, 81.1) in Nepal. Being younger, having rural residents, having lower educational attainment, and being in households with lower wealth quantiles were linked to a higher prevalence of anaemia in most countries.

Conclusion

The findings of this study imply that a variety of individual characteristics play a role in the higher risk of anaemia. To combat anaemia, evidence-based, multidisciplinary policies and initiatives targeting women's health and nutrition, as well as scaling up women's education, empowerment, and socioeconomic position, are needed.

## Introduction

Anaemia affects adolescent girls, women of reproductive age, pregnant women, and children in low- and middle-income countries (LMICs) [1-3]. The WHO estimates that 40% of pregnant women, and one-third of all women of reproductive age are anaemic worldwide, but in South Asia, the prevalence of anaemia among women of reproductive age (15-49 years) is 30% [2,3].

Anaemia occurs when the quantity of red blood cells or the concentration of haemoglobin in them is lower than usual [2]. Anaemia is linked to poor cognitive and motor development in children, as well as decreased work capacity in adults, both of which have an impact on a country's economic development. Several adverse pregnancy consequences are linked to iron deficiency anaemia. Millions of women's health and quality of life may suffer if anaemia is not addressed properly [3]. According to population prevalence, the public health significance of anaemia in a population is classified as follows: not a public health problem: ≤4.9%; mild: 5.0-19.9%; moderate: 20.0-39.9%; or severe: ≥40.0% of the population affected by the problem [4]. Chronic disease, nutritional deficiencies, inflammation, hereditary haemoglobin problems, and socioeconomic status are the primary contributors to anaemia [5].

Despite significant improvements in socioeconomic and health status in most low-income nations in the South and Southeast Asia (SSEA) area, countries continue to face challenges in lowering the high prevalence of malnutrition among women of reproductive age. In fact, progress in reducing anaemia prevalence has been far slower than anticipated, and its socioeconomic cost, particularly throughout low‐ and middle‐income nations, remains a serious problem in the SSEA region [6].

The rising global prevalence of anaemia indicates that, if left untreated, the problem may worsen, notwithstanding current efforts in SSEA countries to improve population health. Understanding the risk factors for anaemia is critical for designing novel and evidence-based strategies to minimise its global prevalence, particularly in developing nations. To develop and implement an evidence-based package of interventions that can generate significant results, successful anaemia reduction programmes must first identify the primary contributing variables. It is vital to collect appropriate knowledge on contextual determinants of anaemia to contribute to the development of timely interventions in anaemia prevention to meet the WHO global nutrition targets 2025 and the nutrition targets of the Sustainable Development Goals 2030. Despite prior attempts to estimate the prevalence of anaemia, few studies have used nationally representative data to explore the prevalence and determinants of anaemia among women of reproductive age in the SSEA region. Hence, the aim of this study is to determine the prevalence of anaemia and the factors that contribute to it among women of reproductive age in 10 SSEA countries.

## Materials and methods

Methods

Data Source, Sampling Technique, and Population

This study belongs to one of our projects under the Demographic and Health Surveys (DHS) programme where we registered our project and got approval (approval number: 161791) to get access and utilise their dataset for the analysis.

Data analysis was undertaken utilising data from 10 SSEA nations' DHS between 2015 and 2021. The included countries were Afghanistan, Bangladesh, Cambodia, India, Indonesia, Maldives, Myanmar, Nepal, Pakistan, and the Philippines. In DHS, a recent population census frame with enumeration areas (EAs) is used as a sampling frame for the first-stage selection. The EAs are selected as primary sampling units or clusters. An EA is a geographical area that serves as a counting unit for the population census and is usually a city block in urban areas or a village in rural areas with 200-300 households [7].

Variables of the study

Dependent Variable

The outcome variable was the prevalence of anaemia, which was determined depending on the women's pregnancy status: if pregnant, a haemoglobin value of less than 11 g/dL is deemed anaemic, and if not pregnant, a haemoglobin value of less than 12 g/dL is considered anaemic [8].

Independent Variables

Age is recorded in completed years and categorised as 15-19, 20-24, 25-29, 30-34, 35-39, 40-44, and 45-49 years. The type of place of residence is the designation of the cluster or enumeration area as an urban area or a rural area. Education is generally reported as the highest level of education attended (not necessarily completed) in categories of no education, primary, secondary, or higher than secondary. Relationship to household head indicated whether the respondent was head of the family, wife, daughter, daughter-in-law, granddaughter, mother, mother-in-law, sister, other relative, or adopted/foster child. Wealth index (WI) was reported in five groups as a background characteristic of the low-income, lower-middle-income, and upper-middle-income countries: poorest, poorer, middle, richer, and the richest. The WI was created based on information about household assets gathered from the household questionnaire. The questionnaire was related to the possession of various consumer goods by the household, such as a car and television, as well as details about the type of drinking water source, flooring, lavatory amenities, and other aspects of the home that were associated with affluence. Rather than being expressed as the quintiles of people at risk for a particular health or population indicator, wealth quintiles were expressed as the quintiles of individuals within the population. For currently pregnant, whether the respondent was currently pregnant at the time of the interview was recorded. Mode of delivery was reported as the percentage of live births delivered by either caesarean section or vaginal/vaginal/instrumental delivery.

Marital status was typically reported as a background characteristic: never married or in union, married, widowed, divorced, no longer living together/separated, and living with a partner. Spouse/partners’ education is generally reported as the highest level of education attended (not necessarily completed) in categories of no education, primary, secondary, higher than secondary, and don’t know. Husband/partner's occupations were categorised under did not work, professional/technical/managerial, sales/services, clerical, agricultural - self-employed/employee, household and domestic, skilled manual/unskilled manual, and others. Employment status was considered as the percent distribution of women aged 15-49 years by whether currently employed, employed in the 12 months preceding the survey but not currently, or not employed in the 12 months preceding the survey [7].

Data management and statistical analysis

For data extraction, additional coding, and both descriptive and analytical analysis, SPSS version 25 software (IBM Corp., Armonk, NY) was utilised. Weighting was used throughout the study to correct for disproportional sampling and non-response, as well as to restore sample representativeness so that the entire sample resembled the country's actual population. We used a weighted sample of 914,496 women of reproductive age (15-49 years) for this investigation. The design weight, which accounts for the sampling units' selection probabilities at different sampling stages, is used to calculate survey weights in DHS surveys [9].

A descriptive analysis was carried out with the use of frequencies and percentages. A multivariate logistic regression model was carried out at a 95% confidence interval (CI). The odds ratio of the independent variables was assessed. To establish the relationship between explanatory variables and anaemia, the correlations test was carried out. Statistical significance was determined by a p-value of less than 0.05 (**p ≤ 0.05).

In underdeveloped nations, national surveys frequently result in partial or inaccurate response reporting. To circumvent these issues, the DHS programme implemented an editing and imputation procedure that yields a data file that precisely represents the population under investigation and can be easily utilised for research. Missing values were displayed when they represent at least 1% of cases in any row in tables with a percent distribution that adds up to 100%. Other unique responses and codes such as “missing”, “inconsistent”, “don’t know”, and “blank” (or "not applicable"), were excluded when calculating statistics; otherwise, they were considered real values [9].

## Results

Characteristics of the study population

This survey comprised 10 SSEA countries. We employed the individual recode (IR) file from DHS data, and a total weighted sample of 914,496 women of reproductive age (15-49 years) was considered (Table 1).

**Table 1:** DHS year and weighted sample among women of reproductive age in 10 South and Southeast Asian countries (n = 914,496). AF: Afghanistan; BD: Bangladesh; CD: Cambodia; IN: India; ID: Indonesia; MD: Maldives; MM: Myanmar; NP: Nepal; PK: Pakistan; PH: Philippines.

AF	BD	CD	IN	ID	MD	MM	NP	PK	PH
2015	2017-2018	2014-2015	2019-2021	2017	2016-2017	2015-2016	2016-2017	2017-2018	2017
29,461	20,127	17,578	724,115	49,627	7,699	12,885	12,862	15,068	25,074

Prevalence and predictors associated with anaemia among women of reproductive age (15-49 years)

**Figure 1:**
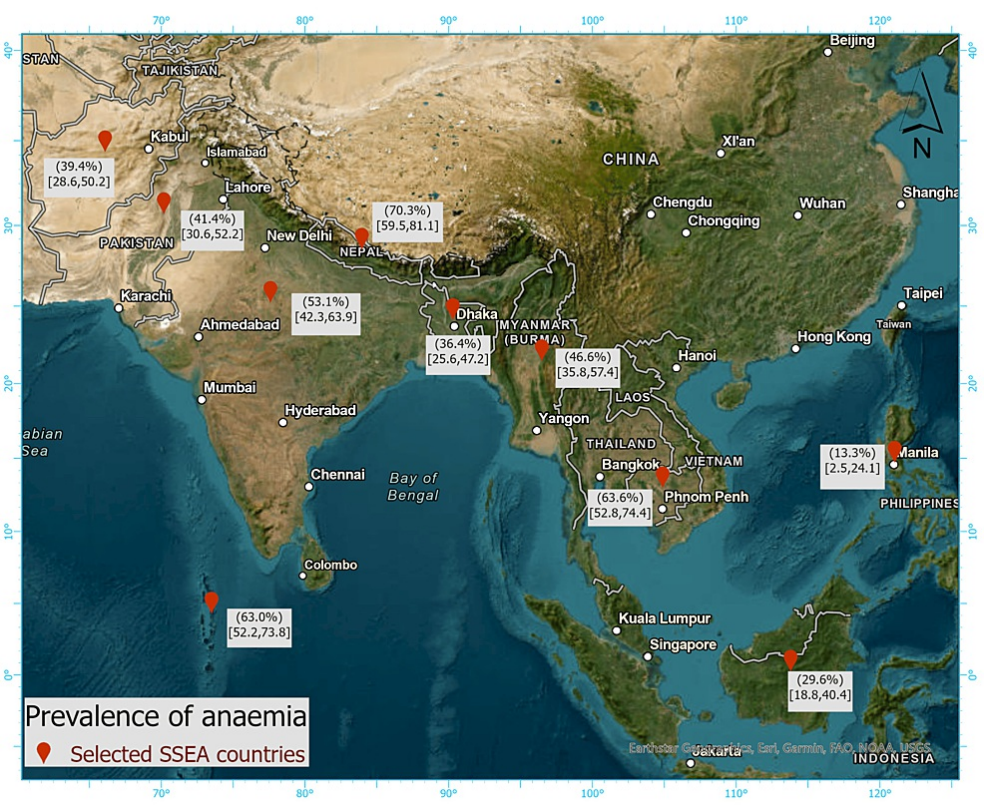
Prevalence of anaemia among women of reproductive age (15–49 years) across selected South and Southeast Asian (SSEA) countries. Image credits: Tahmina Afrose Keya. This image has been created by using ArcGIS Pro software (v3.0.1; Esri, Redlands, CA).

Our study results reveal the prevalence of anaemia among women of reproductive age (15-49 years) across 10 SSEA countries. Overall, 50.17% of women in reproductive age groups were anaemic, ranging from 13.3% in the Philippines to 70.3% in Nepal (Figure 1)*.*

In our study, anaemia was linked to a variety of factors across the selected SSEA countries (Table 2).

**Table 2:** Multivariate logistic regression analysis for determinants associated with anaemia among women of reproductive age (15-49 years) in selected South and Southeast Asian countries. AF: Afghanistan; BD: Bangladesh; CD: Cambodia; IN: India; ID: Indonesia; MD: Maldives; MM: Myanmar; NP: Nepal; PK: Pakistan; PH: Philippines. OR: odds ratio for the logistic regression. * p < 0.001. ^a ^Dependent variable: anaemia prevalence (yes).

	AF	BD	CD	IN	ID	MD	MM	NP	PK	PH
Variables	OR (95% CI)	OR (95% CI)	OR (95% CI)	OR (95% CI)	OR (95% CI)	OR (95% CI)	OR (95% CI)	OR (95% CI)	OR (95% CI)	OR (95% CI)
Anaemia^a^										
Age in years										
15-19	2.2 (2, 1.5)*	1.5 (0.3, 0.5)*	1.8 (1.7, 1.9)*	0.7 (0.7, 1)*	2.4 (2.3, 2.5)*	0.6 (05, 0.7)*	1.0 (0.9, 1.1)	0.5 (0.4, 0.6)*	1.3 (1.1, 1.5)	6.0 (5.5, 6.5)*
20-24	1.6 1.5, 1.7)*	1.6 (0.4, 0.5)*	1.9 (1.7, 2)*	0.8 (0.8, 1)*	2.4 (2.2, 2.5)*	0.5 (0.4, 0.6)*	1.1 (1.0, 1.2)	0.4 (0.4, 0.5)*	1.3 (1.2, 1.4)*	5.7 (5.3, 6.3)*
25-29	1.5 (1.4, 1.6)*	1.6 (0.4, 0.6)*	1.8 (1.7, 1.9)*	0.8 (0.8, 1)*	2.3 (2.2, 2.4)*	0.6 (0.5, 0.6)*	1.2 (1.1, 1.4)*	0.4 (0.3, 0.5)*	1.4 (1.3, 1.5)*	6.5 (5.9, 7.1)*
30-34	1.6 (1.5, 1.7)*	1.8 (0.5, 0.7)*	1.7 (1.6, 1.8)*	0.8 (0.8, 1)*	2.3 (2.2, 2.4)*	0.6 (0.5, 0.7)*	1.1 (1.0, 1.2)	0.4 (0.3, 0.5)*	1.4 (1.3, 1.5)*	7.1 (6.4, 7.8)*
35-39	1.4 (1.3, 1.5)*	1.9 (0.5, 0.7)*	1.5 (1.4, 1.7)*	0.9 (0.9, 1)*	2.4 (2.3, 2.5)*	0.6 (0.5, 0.6)*	1.3 (1.2, 1.4)*	0.4 (0.3, 0.4)*	1.5 (1.4, 1.6)*	6.7 (6.0, 7.4)*
40-44	1.4 (1.3, 1.4)*	1.9 (0.5, 0.7)*	1.7 (1.5, 1.8)*	1 (0.9, 1)	2.4 (2.3, 2.5)*	0.6 (0.5, 0.7)*	1.1 (1.0, 1.2)	0.4 (0.3, 0.4)*	1.4 (1.3, 1.5)*	7.0 (6.2, 7.8)*
45-49	Ref	Ref	Ref	Ref	Ref	Ref	Ref	Ref	Ref	Ref
Residence										
Urban	Ref	Ref	Ref	Ref	Ref	Ref	Ref	Ref	Ref	Ref
Rural	1.5 (1.4, 1.5)*	1.8 (0.6, 0.6)*	1.7 (1.6, 1.7)*	0.8 (0.8, 0.8)*	2.5 (2.4, 2.5)*	0.7 (0.6, 0.8)*	1.1 (1.1, 1.2)*	0.5 (0.4, 0.5)*	1.3 (1.2, 1.3)*	4.6 (4.5, 4.8)*
Educational level										
No education	1.5 (1.4, 1.57),*	2.4 (0.8, 0.9)*	2.5 (2.3, 2.8)*	0.8 (0.8, 1)*	3.5 (3.0, 4.1)*	0.6 (0.5, 0.7)*	1.1 (1.0, 1.2)	0.5 (0.5, 0.6)*	1.5 (1.4, 1.6)*	1.5 (1.2, 1.9)*
Primary	1.6 (1.5, 1.8),*	1.7 (0.5, 0.6)*	1.5 (1.4, 1.6)*	0.8 (0.8, 1)*	3.0 (2.9, 3.1)*	0.6 (0.5, 0.6)*	1.2 (1.1, 1.2)*	0.3 (0.2, 0.3)*	1.0 (0.9, 1.1)	4.0 (3.7, 4.3)*
Secondary	1.6 (1.4, 1.7),*	1.5 (0.4, 0.5)*	1.8 (1.7, 1.9)*	0.9 (0.9, 1)*	2.2 (2.1, 2.3)*	0.6 (0.5, 0.6)*	1.1 (1.0, 1.2)	0.4 (0.3, 0.4)*	1.3 (1.2, 1.4)*	8.2 (7.8, 8.7)*
Tertiary/higher	Ref	Ref	Ref	Ref	Ref	Ref	Ref	Ref	Ref	Ref
Wealth index										
Poorest	2.9 (2.7, 3)*	2.2 (0.7, 0.9)*	1.6 (1.5, 1.7)*	0.8 (0.8, 1)*	3.5 (3.4, 3.7)*	0.7 (0.6, 0.8)*	1.2 (1.1, 1.3)*	1.2 (1.1, 1.3)*	2.4 (2.2, 2.6)*	2.9 (2.7, 3.0)*
Poorer	1.6 (1.4, 1.6)*	2.3 (0.7, 0.9)*	1.9 (1.7, 1.9)*	0.7 (0.7, 1)*	2.3 (2.2, 2.4)*	0.6 (0.5, 0.7)*	1.1 (1.0, 1.2)	0.5 (0.4, 0.5)*	1.7 (1.6, 1.8)*	6.1 (5.6, 6.5)*
Middle	1.2 (1.1, 1.2)*	1.6 (0.4, 0.5)*	1.4 (1.3, 1.5)*	1 (0.9, 1)	2.1 (2.1, 2.2)*	0.8 (0.7, 0.8)*	1.1 (1.1, 1.2)	0.3 (0.3, 0.4)*	1.2 (1.1, 1.3)*	9.1 (8.3, 10.0)*
Richer	1.2 (1.1, 1.2)*	1.5 (0.3, 0.4)*	1.4 (1.3, 1.5)*	1 (0.9, 1)*	2.1 (2.0, 2.2)*	0.4 (0.3, 0.5)*	1.1 (1.0, 1.2)	0.2 (0.2, 0.3)*	1.1 (1.0, 1.2)	12.7 (11.4, 14.2)*
Richest	Ref	Ref	Ref	Ref	Ref	Ref	Ref	Ref	Ref	Ref
Currently pregnant										
No/unsure	1.5 (1.4, 1.5)*	1.8 (0.5, 0.6)*	1.8 (1.7, 1.8)*	0.8 (0.8, 1)*	2.4 (2.3, 2.4)*	0.6 (0.5, 0.7)*	1.1 (1.1, 1.2)*	0.4 (0.4, 0.5)*	1.4 (1.3, 1.4)*	6.6 (6.3, 6.8)*
Yes	Ref	Ref	Ref	Ref	Ref	Ref	Ref	Ref	Ref	Ref
Last mode of delivery										
Vaginal/instrumental delivery	1.7 (1.6, 1.8)	1.5 (0.4, 0.5)*	1.7 (1.6, 1.8)*	0.6 (0.6, 1)*	2.6 (2.5, 2.7)*	0.5 (0.4, 0.6)*	1.2 (1.1, 1.3)*	0.5 (0.4, 0.5)*	1.5 (1.4, 1.6)*	6.3 (5.9, 6.8)*
Caesarean section	Ref	Ref	Ref	Ref	Ref	Ref	Ref	Ref	Ref	Ref
Current marital status										
Never in union/unmarried	Ref	Ref	Ref	Ref	Ref	Ref	Ref	Ref	-	Ref
Married	2.1 (1.8, 2.4)*	1.9 (1.7, 2.3)*	1.7 (1.6, 1.8)*	0.9 (0.9, 1)*	2.4 (2.4, 2.5)*	0.6 (0.5, 0.7)*	1.2 (1.1, 1.2)*	0.4 (0.4, 0.5)*	-	5.3 (5.0, 5.5)*
Widowed	-	-	3.1 (1.9, 4.9)*	-	46.6 (23.1, 93.9)*	0.7 (0.3, 1.6)	-	0.4 (0.1, 2.1)	1.3 (1.1, 1.6)	36.3 (29.9, 43.9)*
Divorced	3.3 (1.8, 6.2)*	2.2 (1.8, 2.8)*	1.7 (1.4, 1.9)*	1 (0.9, 1)*	2.2 (1.9, 2.5)*	0.5 (0.2, 0.9)	1.1 (0.9, 1.4)	0.7 (0.6, 0.9)	1.2 (0.9, 1.7)	3.3 (2.5, 4.4)*
No longer living together/separated	0.9 (0.4, 1.8)	1.5 (1.2, 1.9)	1.6 (1.4, 1.9)*	0.9 (0.8, 1)	2.4 (2.2, 2.7)*	0.6 (0.5, 0.7)*	1.1 (0.9, 1.4)	0.2 (0.1, 0.7)	0.6 (0.4, 1.0)	2.1 (1.1, 4.0)
Living with partner	-	-	4.2 (2.3, 7.7)*	1 (1, 2)*	6.7 (4.3, 10.5)*	0.5 (0.04, 5)	1.8 (1.0, 3.0)	0.2 (0.1, 0.4)*	-	13.0 (9.7, 17.6)*
Husband/partner's highest year of education										
No education	1.9 (1.8, 1.9)*	2.4 (2.2, 2.6)*	2.4 (2.2, 2.7)*	1 (0.9, 1)	5.1 (4.1, 6.3)*	0.6 (0.5, 0.7)*	1.1 (1.0, 1.2)	0.4 (0.3, 0.4)*	1.5 (1.4, 1.6)*	1.5 (1.2, 1.9)*
Primary	1.5 (1.4, 1.6)*	1.7 (1.6, 1.7)*	1.5 (1.4, 1.5)*	1 (0.9, 1)*	3.1 (2.9, 3.2)*	0.6 (0.5, 0.7)*	1.2 (1.1, 1.2)*	0.4 (0.4, 0.5)*	1.2 (1.1, 1.3)*	5.0 (4.6, 5.4)*
Secondary	1.1 (1, 1.1)*	1.4 (1.4, 1.5)*	1.8 (1.7, 1.9)*	0.9 (0.8, 1)*	2.2 (2.1, 2.2)*	0.6 (0.5, 0.7)*	1.2 (1.1, 1.3)*	0.4 (0.3, 0.4)*	1.3 (1.2, 1.4)*	9.4 (8.6, 10.2)*
Higher	Ref	Ref	Ref	Ref	Ref	Ref	Ref	Ref	Ref	Ref
Don't know	2.5 (1.9, 3.2)*	0.5 (0.3, 0.9)	3.3 (2, 5.2)*	0.6 (0.5, 1)	3.5 (1.8, 6.6)*	0.5 (0.4, 0.6)*	1.3 (1.0, 1.7)	0.8 (0.3, 2.0)	0.9 (0.4, 1.8)	-
Husband/partner's occupation										
Did not work	Ref	Ref	Ref	Ref	Ref	Ref	Ref	Ref	Ref	Ref
Professional/technical/managerial	1.2 (1.1, 1.4)*	1.8 (1.7, 2.0)*	2.2 (1.9, 2.4)*	0.9 (0.8, 1)	2.6 (2.4, 2.8)*	0.5 (0.4, 0.6)*	1.2 (0.8, 1.8)	0.4 (0.3, 0.5)*	2.0 (1.9, 2.2)*	9.5 (8.1, 11.1)*
Sales/services		1.7 (1.5, 1.8)*	1.9 (1.4, 2.6)*	0.7 (0.7, 1)*	2.4 (2.2, 2.7)*	0.5 (0.2, 1)	1.0 (0.9, 1.2)	0.1 (0.1, 0.2)*	1.7 (1.4, 2.1)*	20.9 (13.2, 33.2)*
Clerical	1.9 (1.8, 2)*	3.5 (3.1, 4.0)*	1.7 (1.5, 1.9)*	0.7 (0.7, 1)*	2.3 (2.2, 2.5)*	0.6 (0.4, 0.8)*	1.1 (1.0, 1.2)	0.4 (0.4, 0.5)*	1.3 (1.2, 1.4)*	11.0 (8.5, 14.3)*
Agricultural - self-employed/employee		2.4 (2.2, 2.6)*	1.6 (1.5, 1.7)*	1 (0.9, 1)*	2.1 (2.1, 2.2)*	0.9 (0.8, 1.1)	0.9 (0.8, 1.0)	0.5 (0.4, 0.6)*	1.4 (1.2, 1.5)*	3.8 (3.6, 4.1)*
Household and domestic	1.6 (1.5, 1.7)*	1.1 (0.5, 2.4)	2.4 (2.1, 2.7)*	0.8 (0.8, 1)*	2.9 (2.7, 3.0)*	0.6 (0.1, 2.5)	3.3 (1.1, 10.0)	0.3 (0.3, 0.4)*	1.6 (1.4, 1.8)*	15.3 (5.5, 41.9)*
Skilled manual/unskilled manual	1.3 (1.2, 1.4)*	1.2 (1.1, 1.3)*	1.5 (1.4, 1.6)*	0.8 (0.7, 1)*	2.5 (2.4, 2.7)*	0.5 (0.4, 0.7)*	1.5 (1.0, 2.3)	0.5 (0.4, 0.6)*	1.4 (1.3, 1.5)*	9.6 (7.9, 11.7)*
Others	1.7 (1.6, 1.9)*	0.8 (0.3, 2.1)	1.6 (1.1, 2.4)	0.8 (0.7, 1)*	2.7 (2.0, 3.5)*	0.1 (0.01, 0.5)	1.3 (1.1, 1.4)*	0.4 (0.2, 0.6)*	1.0 (0.9, 1.1)	11.0 (9.8, 12.3)*
Respondent currently working										
No	Ref	Ref	Ref	Ref	Ref	Ref	Ref	Ref	Ref	Ref
Yes	2.6 (2.4, 2.8)*	2.4 (2.3, 2.6)*	1.6 (1.5, 1.6)*	1 (0.9, 1)*	2.3 (2.3, 2.4)*	0.6 (0.5, 0.7)*	1.1 (1.1, 1.2)*	0.5 (0.4, 0.5)*	1.1 (1.0, 1.2)	8.0 (7.6, 8.5)*

In Afghanistan and Cambodia, younger women (<35 years old) were more likely to be anaemic. In Bangladesh, India, Indonesia, Myanmar, Pakistan, and the Philippines, however, older age groups (>35 years) were more likely to be anaemic. Rural women in Bangladesh and Indonesia were more likely to be anaemic than their counterparts in SSEA. In Bangladesh, Cambodia, and Indonesia, women with no education were more likely to be anaemic compared to those with a higher degree of education. Women with a secondary education were more likely to be anaemic in the Philippines. Women with a higher education, on the other hand, were more likely to be anaemic in Afghanistan and Myanmar.

Women who had poorer wealth status were more likely to be anaemic compared to women with richer wealth status in Afghanistan, Bangladesh, India, Indonesia, Myanmar, Nepal, and Pakistan, but were less likely to be anaemic in Cambodia and the Philippines. Non-pregnant women in the SSEA had a higher risk of anaemia than their counterparts in Bangladesh, Cambodia, Indonesia, and the Philippines. Women who had a history of vaginal/instrumental delivery were more likely to be anaemic than their counterparts in Afghanistan, Cambodia, Indonesia, and Pakistan. Among the women from Bangladesh, India, Myanmar, and the Philippines, however, caesarean section was a substantial predictor of anaemia. In Afghanistan, Bangladesh, Cambodia, India, Indonesia, Myanmar, and the Philippines, women who were widowed/divorced/separated/living with a partner were more likely to be anaemic than women who had never married.

In most of the selected SSEA countries, women with a less educated spouse or partner (tertiary/higher education level) were more likely to be anaemic except for Pakistan. In the Maldives and Nepal, however, spouse/partners’ education was not a major predictor. Women whose spouses/partners worked in agricultural-self-employed/employee and skilled manual/unskilled manual/sales/services/clerical/domestic occupations were more likely to be anaemic in Afghanistan, Bangladesh, India, Myanmar, and the Philippines than women whose husbands/partners did not work. Furthermore, women whose spouses/partners worked in professional/technical/managerial jobs were more likely to be anaemic in Pakistan. Working women in Afghanistan, Bangladesh, India, and the Philippines were more likely to be anaemic than their counterparts (Table 2).

**Figure 2:**
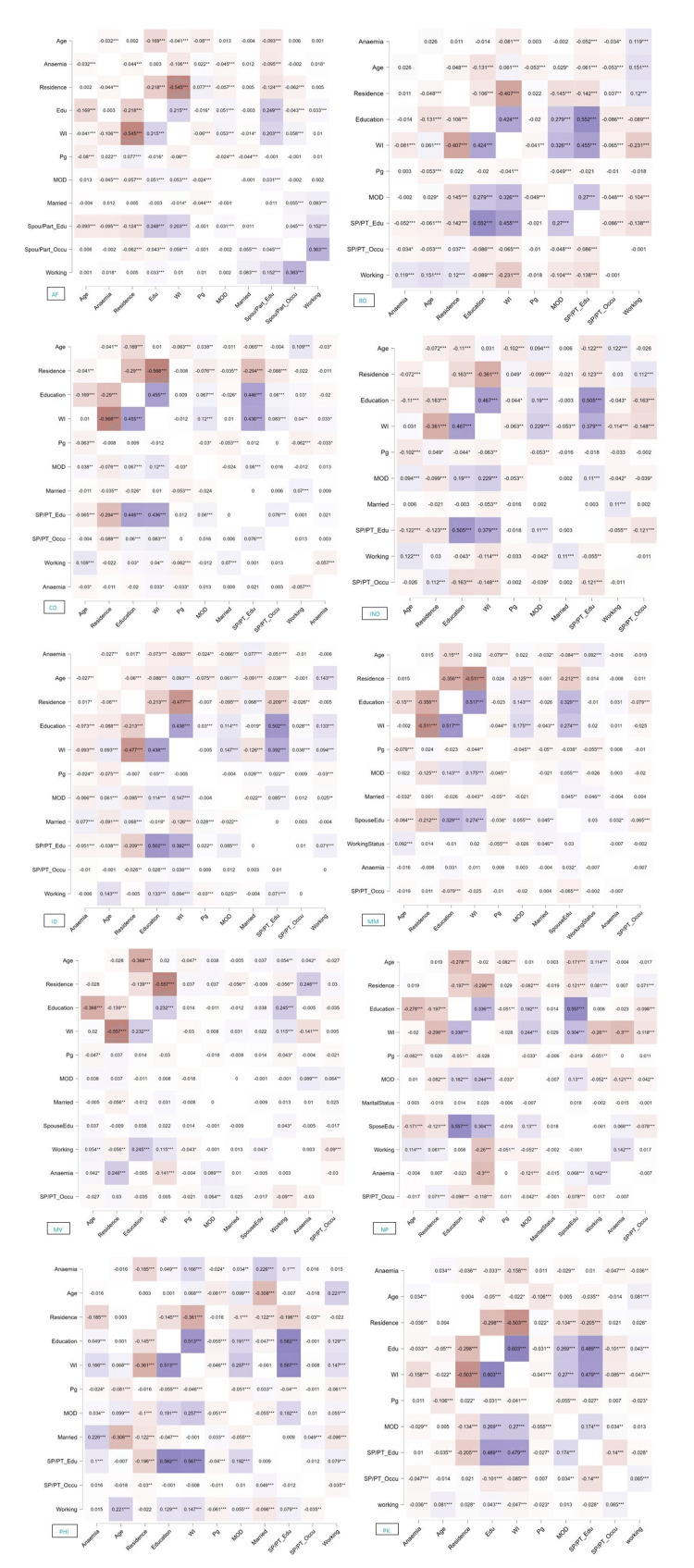
Pearson's correlations. Conditioned on variables: Anaemia_P_Yes *** p < 0.001 = statistically significant. AF: Afghanistan; BD: Bangladesh; CD: Cambodia; IN: India; ID: Indonesia; MD: Maldives; MM: Myanmar; NP: Nepal; PK: Pakistan; PH: Philippines; WI: wealth index; MOD: mode of delivery.

According to the study, there was a significant correlation between a woman's WI and the educational status of her spouse or partner in Afghanistan. Concurrently, spouses/partners’ education and occupational attainment were also significantly correlated with their wives’ working conditions. Women's anaemic status and working conditions were significantly correlated in Bangladesh. Alongside, there was a strong correlation between their age and residential status and their working conditions. In Cambodia, the WI was strongly correlated with their education and significantly correlated with their mode of delivery. Simultaneously, there was a significant correlation between their mode of delivery and education, WI, and the education of their spouses or partners. In India, there was a significant correlation between women's educational attainment and the mode of delivery. In Indonesia, a significant correlation between a woman's anaemia and her married status was found (p < 0.001). In Myanmar, both the educational status of the women and that of their spouses or partners were correlated to the anaemia of the women. Women likewise had a substantial correlation between age, working status, and marital status. In the Maldives, a significant relationship existed between a woman's anaemia and her residential status and mode of delivery. Women’s working status and the educational level of their spouse or partner were found to be substantially correlated with their anaemic condition in Nepal. In the Philippines, the anaemic condition of women was significantly correlated with their marital status, WI, and the educational level of their husband or partner. Age and women's anaemia were substantially connected in Pakistan. Besides, there was a strong correlation between women's WI, education, delivery method, and partner's or spouse's educational attainment (Figure 2).

## Discussion

This study looked at the prevalence of anaemia and its determinants in 10 SSEA countries. In this study, the prevalence of anaemia was determined to be 50.17%, with rates ranging from 13.3% in the Philippines to 70.3% in Nepal. When anaemia prevalence is greater than 40% in any population, it is considered a serious public health issue [3]. In our study, anaemia was found to be a serious public health problem in six SSEA countries. Dietary habits, nutritional deficiencies, particularly iron, vitamin, and folic acid, chronic inflammation, parasitic infections, geographical and cultural variables, and genetic haemoglobin abnormalities in these regions could all be contributors [10].

Chaparro and Suchdev found that anaemia affected 52.5% of reproductive-age women in SSEA. Because six of the 10 nations in this SSEA study with a bigger representation showed a relatively higher frequency of anaemia among women of reproductive age, we assume that these findings are congruent with our findings. Furthermore, anaemia prevalence in the rest of the selected countries also showed significant public health issues (>20%). In the SSEA countries, women with poor socioeconomic levels, lack of sanitation and water sources, poverty, lack of education, and gender inequality were all significant predictors of anaemia [10].

Individual factors such as age, residence, education, pregnancy, mode of delivery, smoking habit, marital status, economic status, working status, and husbands'/partners' education and occupation were found to be strong predictors of anaemia among women in the reproductive age groups of the selected SSEA countries in the current study. These findings are also in line with findings from other LMIC studies that revealed similar drivers of anaemia among reproductive-age women [11]. Balarajan et al. found that education level, wealth, and individual behaviours were all major contributors to anaemia among women in a systematic analysis done across low-income countries [12]. In our study, women's age was found to be a significant predictor of anaemia. In most of the countries studied, women over 35 years of age were more likely to be anaemic. One study found a similar pattern of connections between age and anaemia, with women aged 40-49 years experiencing the highest levels of anaemia [10]. The possible reason could be explained by nutritional deficiencies, increased parity and gravidity, and chronic illnesses among women in those nations [13]. These findings contrast with anaemia-age trends calculated by Kassebaum et al. (2018) who found a consistent drop in anaemia prevalence in women above the age of 20 years. In the current study, however, 35-year-olds were more likely to be anaemic in Afghanistan and Cambodia [14]. The detrimental effect of lower dietary iron intake and the increased demand for iron imposed by iron loss during menstruation, adolescent pregnancy, and lactation could explain the higher prevalence of anaemia among relatively younger women [14,15].

Apart from Bangladesh and Indonesia, women from rural areas were less likely to suffer from anaemia. These findings are consistent with the findings of other studies [16]. High rates of infectious infections and parasitic infestations, limited access to medical care, and a lack of health and nutrition information among women in these LMCIs could all contribute to the risk of becoming anaemic. Dietary limitations, as well as a poor intake of nutrient-dense foods and hereditary diseases, on the other hand, may be contributing to the higher prevalence of anaemia among urban women [17].

In most of the SSEA countries except Afghanistan and Myanmar, those with a lower level of education (<tertiary level) were more likely to be anaemic. This finding is in line with prior research, which revealed that women with a higher education were less likely to be anaemic [18]. This can perhaps be explained by that women with a higher level of education were less likely than their counterparts to have poor eating habits. Low socioeconomic status, lack of awareness, and cultural and attitudinal variations, on the other hand, could all be the possible reasons for anaemia among higher educated women in Afghanistan and Myanmar [19].

Women with lower wealth status were more likely to be anaemic in most of the SSEA nations studied, except for Cambodia and the Philippines. However, other studies reported the opposite relationship between socioeconomic status (SES) and anaemia prevalence [20]. A high rate of infectious diseases and parasitic infestations, restricted access to health care, high parity, a short birth interval, a poor diet in terms of quantity and quality, and a lack of health and nutrition awareness could all be plausible explanations. Cambodian diet habits, as well as hereditary haemoglobin disorders, could explain the reasons. Furthermore, a higher prevalence of anaemia among the Philippines' wealthiest women could be due to multiparity and teenage pregnancy [21].

In most of the countries studied, non-pregnant women were more likely to be anaemic. Micronutrient deficits, haemoglobinopathies, and viral and parasitic disorders may all have a role [17]. Other research, however, found the opposite [22]. This is likely due to an increase in iron demand during pregnancy, insufficient antenatal visits, or non-compliance with iron supplementation during pregnancy [22]. Unmarried women and women who had never had sexual experience previously were less likely to be anaemic in most of the SSEA countries in our study. This finding is consistent with the evidence from other studies conducted in various settings [23]. This might be because of the married/widowed/divorced/separated/living with partner women working outside in addition to caring for children and managing home obligations [23].

In most of the SSEA countries, women with a less educated spouse/partner were more likely to be anaemic. This finding was consistent with some previous research [24]. Low decision-making skills of women to access available resources and health information, husband's illiteracy, poverty, and gender inequity in LMICs could be the reasons [12]. Regardless of whether their spouses were employed, most women from SSEA countries, except for Cambodia and Indonesia, were more likely to be anaemic in our study. Other research in different settings has found similar results [25]. The poorest households, less-educated women, and women living in rural areas in Cambodia and Indonesia, however, might be the most likely explanation for the opposite findings [26].

Being currently working was a determinant for a lower prevalence of anaemia among women of reproductive age in most of the selected SSEA countries, which is consistent with studies conducted in different settings across the LMICs [11,23]. This could be due to low decision-making autonomy regarding health care and experience of intimate partner violence (IPV) as well as low socioeconomic status and inadequate dietary intake [22]. Working women from Afghanistan, Bangladesh, India, and the Philippines, on the other hand, were more likely to be anaemic in our study. These findings are in line with those of a study conducted in the Philippines [27,28]. A lack of thorough awareness about anaemia, as well as low utilisation of healthcare facilities, could be the reasons [21,24].

Anaemia among women of reproductive age was significantly positively related to their age, marital status, WI, residential status, mode of delivery, working status, and the educational attainment of their husband or partner in most of the selected SSEA countries (p < 0.001). Alongside, there was a strong correlation between the explanatory variables in most of the selected countries. This relationship was also observed in a meta-analysis where higher educational attainment, highest wealth quintile, residence in urban areas, and increased maternal age were found also protective against the risk of anaemia [23].

We were unable to establish a cause-and-effect relationship between the predictors and the outcome variables because of the cross-sectional character of the DHS data. Furthermore, our research was limited to the 10 countries of SSEA. Despite these drawbacks, the study's strengths included a multicounty analysis with a large sample size and competent statistical analysis that considered the hierarchical nature of DHS data. Hence, policymakers and programme administrators can use more accurate and generalizable data to design individual-level intervention plans. Moreover, our study underlines the necessity for a comprehensive intervention and the development of initiatives to raise awareness of the detrimental effects of anaemia among women. Only a few anaemia research studies have been carried out in SSEA, and they were all small-scale and limited to a single location.

## Conclusions

The prevalence of anaemia among women of reproductive age is a serious public health issue in the SSEA region, according to this study. Multiple factors at the individual level have been linked to the development of anaemia. To combat the high prevalence of anaemia among women of reproductive age across the SSEA countries, appropriate interventions should be considered in upgrading programmes and policies that focus on improving socioeconomic status, women's education, and empowerment, effective health education, quality healthcare facilities equally in rural and urban areas, and reducing gender disparity.

It is critical to pay special attention to the unique circumstances that contribute to women's vulnerability. Particular attention should be paid to overcoming low socioeconomic positions. To meet the sustainable development goals and targets for women's health, existing national policies and programmes must be updated based on new evidence.
